# Magnetic Resonance Imaging Findings of Knees and Spines in Recreational Runners who Completed 1000 Marathons

**DOI:** 10.7759/cureus.6382

**Published:** 2019-12-14

**Authors:** Hyechang Rhim, Young Ha Kim, Myung Gyu Kim, Ki-Mo Jang, Seung Woo Suh

**Affiliations:** 1 Medicine, Korea University College of Medicine, Seoul, KOR; 2 Orthopaedics, Korea University Anam Hospital, Seoul, KOR; 3 Radiology, Korea MS Diagnostic Radiology Clinic, Seoul, KOR

**Keywords:** marathon running, running, knee, spine, mri, joints

## Abstract

Introduction

Marathon running is a popular recreational activity, but the effects of chronic running on the knee or other joints remain unclear. The purpose of this article was to evaluate any degenerative changes in the knees and spines of recreational runners who completed at least 1000 marathons.

Methods

Recreational runners who completed at least 1000 marathons were recruited. Magnetic resonance imaging (MRI) of both knees and spines of six such runners was performed with a 1.5 T MR scanner. The anatomical structures of the knee joint including meniscus, bone marrow, cartilage, ligaments, and joint effusion were examined along with other abnormalities. Spinal alignment, degenerative change in intervertebral disc, intervertebral disc herniation, osteoarthritis in facet joint, degenerative anterior/lateral spur, and other abnormalities were evaluated.

Results

In terms of knee joints, one runner showed degeneration at the meniscus, while three runners had cartilage lesions. However, none of the six runners showed radiologic evidence of knee osteoarthritis. All six runners demonstrated degenerative changes in intervertebral spinal discs.

Conclusions

Running 1000 marathons may not have a harmful effect on the knee joints and may not result in osteoarthritis. However, it is unclear whether degenerative changes in the spine are derived from running or aging.

## Introduction

Marathon running is a popular recreational activity, performed by more than 30 million people in the United States every year [1]. However, a systematic review demonstrated that the incidence of lower-extremity running injuries ranges from 19.4% to 79.3%, and the most common site of such injuries was the knee [2]. As the onset and progression of osteoarthritis (OA) are heavily affected by cartilage lesions, it is worthwhile to determine whether knee injury from running can ultimately lead to OA [3]. The effect of running on degenerative disease is still controversial, and magnetic resonance imaging (MRI) techniques have been used to quantitatively and qualitatively evaluate articular cartilage, meniscus, and marrow changes. A systematic review of studies using MRI before and after long-distance running showed that athletes without risk factors for the degenerative joint disease may present temporary but not irreversible harmful effects in a knee joint [4]. However, the effects of chronic running on knee or other joints remain unclear. The authors hypothesized that marathon running at a slow pace would not be detrimental to knee and spine joints. Therefore, the purpose of this study was to evaluate any degenerative changes in the knees and spines of unique recreational runners who completed at least 1000 marathons.

## Materials and methods

Subject population and demographics

This study included six male recreational runners who completed at least 1000 marathons. Informed consent was obtained from all individual participants included in the study. Information on age, height, weight, training distance, intensity, number of marathons completed, medical and surgical history, and past running-related injuries were collected. 

MRI data acquisition

MRI of both knees and spine was performed with 1.5T MRI scanner. This MR scanner called Siemens Magnetom Avanto was manufactured by Siemens Healthineers and designed for various settings including orthopedics and examination of ligament tears to cartilage degeneration. The following sequences were obtained for both knees: Axial fat suppressed (FS) turbo spin echo (TSE) proton density weighted imaging (PD-WI; repetition time [TR] 2800 ms, echo time [TE] 31 ms, slice thickness 4 mm, slice gap of 0.30, matrix 384 x 269), coronal FS TSE PD-WI (TR 2800 ms, TE 31 ms, slice thickness 4 mm, slice gap of 0.20, matrix 384 x 269), coronal T1-WI (TR 757 ms, TE 12 ms, slice thickness 4 mm, slice gap of 0.20, matrix 448 x 314), sagittal TSE PD-WI (TR 2500 ms, TE 27 ms, slice thickness 4 mm, slice gap of 0.20, matrix 448 x 314), sagittal FS TSE PD-WI (TR 3290 ms, TE 32 ms, slice thickness 4 mm, slice gap of 0.30, matrix 384 x 307), sagittal FS three-dimensional SPACE (Sampling Perfection with Application optimized Contrasts using different flip angle Evolution; TR 1200 ms, TE 33 ms, slice thickness 0.8 mm, no intersection gap, matrix 256 x 258). The following sequences were obtained for the spine: sagittal TSE T2-WI (TR 3020 ms, TE 87 ms, slice thickness 4 mm, slice gap of 0.35, matrix 448 x 269), sagittal T1-WI (TR 635 ms, TE 11 ms, slice thickness 4 mm, slice gap of 0.35, matrix 448 x 291), axial TSE T2-WI (TR 3300 ms, TE 88 ms, slice thickness 4 mm, slice gap of 0.30, matrix 320 x 256), axial T1-WI (TR 618 ms, TE 11 ms, slice thickness 4 mm, slice gap of 0.30, matrix 320 x 256).

Image analysis

The anatomical structures of the knee joint including meniscus, bone marrow, cartilage, ligaments, and joint effusion along with other abnormalities were examined by an orthopedic surgeon specialized in knee and a radiologist with 25 years of experience.

Meniscal lesions were scored using a five-point scale (grades 0-4). Grade 0 represented normal meniscus. A grade 1 lesion was characterized by punctate signal intensities not contiguous with an articular surface. A grade 2 lesion represented linear intrameniscal intensity without articular surface extension. A grade 3 lesion indicated meniscal tear characterized by signal intensity extended to one articular surface of the meniscus. A grade 4 lesion was defined as a signal intensity reaching the upper or lower surface of one meniscus (complex meniscal tear) [5-6].

The Outerbridge classification system was used to score cartilage lesions on MR images (grades 0-4). A grade 1 lesion was characterized by an irregular signal of the cartilage matrix but with an intact surface. A grade 2 lesion represented shallow superficial ulceration, fibrillation, or fissuring involving less than 50% of the depth of the articular surface, while a grade 3 lesion indicated the involvement of 50% or more of the depth of the articular cartilage. A grade 4 lesion was characterized by full-thickness chondral wear with exposure of subchondral bone [7].

According to a previous study, bone marrow edema was scored on a three-point scale, ranging from 0 to 2. A grade 1 lesion was defined as signal hyperintensities on short-T1 inversion recovery (STIR) images less than 10 mm in maximum diameter, while a grade 2 lesion represented signal hyperintensities beyond 10 mm in maximum diameter [8].

Ligaments, including the anterior and posterior cruciate ligaments and the medial and lateral collateral complexes, were scored on a three-point scale ranging from 0 to 2. A grade 1 lesion indicated partial tear, while a grade 2 lesion represented complete tear. Joint effusion was graded as 0 if only minimal joint effusion was present on axial T2 and sagittal dual TSE. Fluid collection greater than 1 cm in sagittal diameter in the retropatellar bursa was graded as 1. In addition, other abnormalities such as iliotibial band syndrome, bursal fluid collection, and signal alteration or thickening of the patellar tendon were recorded [9].

Spinal alignment, degenerative change in the intervertebral disc, intervertebral disc herniation, osteoarthritis in facet joint, degenerative anterior/lateral spur, and other abnormalities were examined by an orthopedic surgeon specialized in the spine and the same radiologist who evaluated the knees.

## Results

Subject population and demographics

The average age of these participants was 61 years, with the youngest runner being 55 and the oldest being 68. Running history ranged from 12 to 19 years. Body mass index (BMI) was calculated using weight and height. The average BMI for these runners was 24. The slowest marathon finishing time was six hours 58 minutes, while the fastest time was two hours 56 minutes. The average finishing time for these six runners was four hours 17 minutes. Their weekly mileage ranged from 80km to 180km, which was mostly composed of slow jogging three to four times a week. Four subjects were rearfoot runners, while the other two were midfoot and forefoot runners. All but one runner had suffered running-related injuries in the past, but all had been injury free for at least six months prior to MRI. The characteristics of subjects are summarized in Table 1.

**Table 1:** The characteristics of subjects included in the study hr, hours; min, minutes; sec, seconds; MRI, magnetic resonance imaging; BMI, body mass index

	Subject 1	Subject 2	Subject 3	Subject 4	Subject 5	Subject 6
Age	56	65	56	62	70	65
Gender	Male	Male	Male	Male	Male	Male
Height (cm)	169	179	177	173	167	164
Weight (kg)	65	74	82	70	68	66
BMI	22.76	23.1	26.17	23.39	24.38	24.54
Year of starting running marathons	1995	2004	2005	2005	2001	2005
Year of completing 1000 marathons	2014	2018	2017	2017	2016	2018
Fastest marathon time (hr:min:sec)	2:56:59	3:10:00	3:28:24	3:18:00	03:28:07	03:18:09
Slowest marathon time (hr:min:sec)	6:58:02	5:30:00	5:55:47	5:20:00	05:12:00	05:59:04
Average marathon time (hr:min:sec)	4:20:00	4:00:00	4:35:26	4:00:00	04:31:27	04:20:00
Running-related injuries	Ankle, Knee, Foot, Left hip	Plantar fasciitis Stress fracture	Plantar fasciitis	Left knee	Knee pain, spine	None
Period off-running	1997, 2016-present for personal reasons	6 months dues to above injuries	2017 for 6 months due to above injury	None	None	None
Any injury six months prior to MRI	None	None	None	None	None	None
Weekly Mileage	0~120km, variable	150~180km	100km	80~120km	200km	150km
Training Intensity	Mostly jogging	Mostly jogging (3 marathons/week)	Mostly jogging	Mostly jogging	Mostly jogging (5~6 marathons/week)	Mostly jogging
Past disease history	None	Hypertension	None	None	Prostate cancer	None
Footstrike pattern	Rearfoot	Midfoot	Rearfoot	Forefoot	Rearfoot	Rearfoot

Knee

Five runners showed no abnormality in the meniscus. The other had grade 3 degeneration at the body of the medial meniscus in both knees. However, no functional impairment was seen in this runner.

Among the six runners, three showed cartilage lesions, one each with grade 2 or 3 lesions in the mid-segment of the medial femoral condyle in both knees. The other runner had grade 1 cartilage lesions in the mid-segment of the medial femoral condyle in both knees.

All participants with cartilage lesions showed grade 1 bone marrow edema in similar regions of the medial femoral condyle. These runners were temporarily symptomatic during and after running marathons.

None of the participants showed knee joint effusion or abnormality in ligaments. Two runners suffered from iliotibial band syndrome, both of whom were symptomatic during and after marathons and relieved after rest. One runner had bilateral tenosynovitis in semitendinosis and gracilis tendons. Another runner showed chondromalacia in the right patella and occasionally complained of pain in front of the right knee.

Spine

Four runners lost normal lordosis, and none of the runners showed scoliosis. All runners demonstrated degenerative changes in intervertebral discs, and four runners had intervertebral disc herniation. Five runners showed some degree of osteoarthritis in face joints, and four runners had degenerative anterior/lateral spurs. One runner demonstrated spondylolytic spondylolisthesis, and another showed bilateral spondylolysis. Despite these radiologic findings, all runners were clinically asymptomatic.

The results of knees and spines of all six runners are summarized in Tables 2 and 3, respectively.

**Table 2:** The characteristics of knee lesions found in individual subject NO., number; BM, bone marrow; B/L, bilateral

Subject No.	Meniscus		Cartilage		BM Edema		Ligaments		Joint effusion		Others
	Right	Left	Right	Left	Right	Left	Right	Left	Right	Left	
1	0	0	1	1	1	1	0	0	0	0	(L) Iliotibial band syndrome
2	3	3	0	0	0	0	0	0	0	0	Tenosynovitis in (B/L) semitendinous tendon (L) gracilis tendon (L) bipartite patella
3	0	0	2	2	1	1	0	0	0	0	(B/L) Subchondral cysts
4	0	0	0	0	0	0	0	0	0	0	(R) Patella: Grade 1 chondromalacia
5	0	0	3	3	1	1	0	0	0	0	N/A
6	0	0	0	0	0	0	0	0	0	0	(L) Iliotibial band syndrome

**Table 3:** The characteristics of spine abnormalities found in individual subject OA, osteoarthritis

Subject No.	Alignment	Degenerative change in intervertebral disc	Intervertebral disc herniation	OA in facet joint	Degenerative anterior/lateral spur	Others
1	Loss of normal lordosis No scoliosis	L3-L4, L4-L5, L5-S1.	Small central HNP at L4-L5.	Mild OA at L3-L4, L4-L5	No spur	N/A
2	Loss of normal lordosis No scoliosis	L4-L5, L5-S1	No HNP	L4-L5, L5-S1	L4-L5, L5-S1	Spondylolytic spndylolisthesis of grade I with both foraminal stenosis at L5-S1. Mild degenerative retrolisthesis at L4-L5
3	Normal lordosis No scoliosis	L3-L4, L4-L5, L5-S1.	No HNP	No OA	No spur	N/A
4	Loss of normal lordosis No scoliosis	L3-L4, L4-L5, L5-S1	diffuse disc bulge at L3-L4, L4-L5, L5-S1	Mild OA at L3-L4, L4-L5, L5-S1	Small spur at L3-L4, L4-L5, L5-S1	Bilateral spondylolysis at L5.
5	Loss of normal lordosis No scoliosis	L2-L3, L3-L4, L4-L5, L5-S1	Small central HNP at L4-L5	L1-L2, L2-L3	Small spur at L2-L3, L3-L4	N/A
6	Normal lordosis No scoliosis	T11-T12, T12-L1, L1-L2, L2-L4, L3-L4, L4-L5, L5-S1.	Diffuse disc bulge at L5-S1	L1-L2,L2-L3, L3-L4, L4-L5	Spur at L1-L2	N/A

## Discussion

Many studies have reported acute MRI findings of the knee before and after long distance running, but few studies have investigated the chronic impact of long-distance running on human joints. In the present study, we used MRI to evaluate any degenerative changes of knees and spines in runners who completed at least 1000 marathons, which may be considered excessive running. 

Among six runners, one had a grade 3 meniscus lesion. A previous study showed that the prevalence of meniscal tears in marathon runners was no higher than that reported for sedentary persons, and the runners had the same amount of meniscal degeneration as the nonrunners [10]. Also, another study found that incidental meniscal findings on MRI of the knee are common in the general population and increase with age [11]. Thus, the grade 3 meniscus lesion seen in this runner might be an incidental finding of an age-related lesion rather than a result of excessive running.

Three runners had cartilage lesions ranging from grade 1 to 3, all of which were accompanied by mild bone marrow edema. A previous study reported cartilage degeneration of individuals with no history of knee pain or injury or joint injury on MRI, and another study found a high prevalence of tibiofemoral cartilage defects in non-running, asymptomatic, middle‐age people [12-13]. Despite the lesions found in MRI, three runners in our study reported temporary symptoms rather than chronic pain. These cartilage lesions, like the meniscus lesion found in another runner, may be age-related rather than a result of excessive running because the two other runners had radiologically normal joint architecture. The heterogeneous radiologic findings in our study suggest that running 1000 marathons may not necessarily have a detrimental effect on knee joints.

Interestingly, none of the runners showed radiologic findings of osteoarthritis including asymmetric joint space narrowing, subchondral sclerosis, osteophyte formation, or subluxation [14]. This result contrasts with a previous study that concluded that high levels of physical activity may be a risk factor for symptomatic osteoarthritis [15]. Indeed, a previous study showed that running a single marathon causes only subtle lesions. The authors stated that the musculoskeletal system adapts to the repetitive external impact loading in marathon runners [8]. Our result suggests that the body may be able to adjust to running 1000 marathons at a recreational pace, which therefore may not be a direct risk factor for osteoarthritis. Our finding partially supports the result of a meta-analysis showing that recreational runners have a lower occurrence of osteoarthritis compared with competitive runners or controls [16]. If using a recreational running pace, running 1000 marathons may not cause the knee damage previously expected.

Unlike the heterogeneous MRI findings in the knees, the spines of all six runners demonstrated degenerative changes in intervertebral discs. All but one runner showed osteoarthritis in facet joints. Compared to ample MRI studies investigating the impact of running on knees, there are few studies examining spines. Previous studies have shown disc degeneration in 75% of athletes versus 31% in non-athletes [17-18]. Although the runners in this study are not elite, their duration and volume of running may resemble those of the elite athletes. The findings of runners in our study may be explained by a study that noted intervertebral disc changes after one hour of running, especially at the L5-S1 [19]. All runners in our study demonstrated disc degeneration, with common involvement of the L5-S1 levels, along with other varied levels. Due to long-term, long-distance running, loading on the spine may have accumulated and caused this damage. Impact loading is produced during the landing phase of running, in which impacts are transmitted from the legs to the spine through repeated landings [20]. Running inflicts a ground reaction force of two to three times the body weight to the spine [21]. Due to this impact loading, the spine may be more vulnerable to running than knee joints, which are minimally affected by a single marathon [8]. Even 30 minutes of moderate-intensity running caused intervertebral disc compression in young adults, and running 1000 marathons cumulatively resulted in spinal lesions in our runners. However, as the prevalence of disc degeneration in this age population (>50 years) is relatively high (>90%), age-related degeneration may have contributed to the findings seen in these runners [22]. Furthermore, a previous study found that about 57% of individuals 60 years or older who had never had low-back pain, sciatica, or neurogenic claudication may show a substantial abnormality on spinal MRI [23]. Even though all runners in our study showed spine abnormalities, the clinical relevance of these findings is questionable since the runners were asymptomatic.

The main limitation of our study is the lack of baseline evaluations on the knees and spines of runners. Therefore, we cannot determine whether degenerative changes and osteoarthritis of facet joints seen in spines resulted from running or normal aging. Unlike the spine, none of the runners showed evident degenerative changes in the knees. Due to its irreversible nature, osteoarthritis absent at the time of MRI can be assumed to not be present at baseline as well, indicating that running does not cause osteoarthritis of the knee joint. Considering that age is a risk factor for knee osteoarthritis, running may have had a protective effect on these runners. However, due to the small sample size, the findings may not be generalized to the normal population. Moreover, due to the lack of baseline evaluations, we could not conclude whether meniscal and cartilage lesions derived from running or aging.

## Conclusions

The results of our study show that running 1000 marathons may not have harmful effects on knee joints and may not result in knee osteoarthritis. However, spinal MRI in these runners showed degenerative changes, all of which involved L5-S1 levels. It remains unclear whether these findings are running-related or age-related. Further studies are needed to evaluate the chronic effect of running on knee and spine joints. 

## References

[1] Hohmann E, Wortler K, Imhoff AB (2004). MR imaging of the hip and knee before and after marathon running. Am J Sports Med.

[2] van Gent RN, Siem D, van Middelkoop M, van Os AG, Bierma-Zeinstra SM, Koes BW (2007). Incidence and determinants of lower extremity running injuries in long distance runners: a systematic review. Br J Sports Med.

[3] Behzadi C, Welsch GH, Laqmani A (2016). The immediate effect of long-distance running on T2 and T2* relaxation times of articular cartilage of the knee in young healthy adults at 3.0 T MR imaging. Br J Radiol.

[4] Hoessly ML, Wildi LM (2017). Magnetic resonance imaging findings in the knee before and after long-distance running-documentation of irreversible structural damage? A systematic review. Am J Sports Med.

[5] Stoller DW, Martin C, Crues JV, 3rd 3rd, Kaplan L, Mink JH (1987). Meniscal tears: pathologic correlation with MR imaging. Radiology.

[6] Kocabey Y, Tetik O, Isbell WM, Atay OA, Johnson DL (2004). The value of clinical examination versus magnetic resonance imaging in the diagnosis of meniscal tears and anterior cruciate ligament rupture. Arthroscopy.

[7] Outerbridge RE (1961). The etiology of chondromalacia patellae. J Bone Joint Surg Br.

[8] Schueller-Weidekamm C, Schueller G, Uffmann M, Bader TR (2006). Does marathon running cause acute lesions of the knee? Evaluation with magnetic resonance imaging. Eur Radiol.

[9] Schueller-Weidekamm C, Schueller G, Uffmann M, Till B (2006). Incidence of chronic knee lesions in long-distance runners based on training level: findings at MRI. Eur J Radiol.

[10] Shellock FG, Deutsch AL, Mink JH, Kerr R (1991). Do asymptomatic marathon runners have an increased prevalence of meniscal abnormalities? An MR study of the knee in 23 volunteers. AJR Am J Roentgenol.

[11] Englund M, Guermazi A, Gale D, Hunter DJ, Aliabadi P, Clancy M, Felson DT (2008). Incidental meniscal findings on knee MRI in middle-aged and elderly persons. N Engl J Med.

[12] Beattie KA, Boulos P, Pui M, O'Neill J, Inglis D, Webber CE, Adachi JD (2005). Abnormalities identified in the knees of asymptomatic volunteers using peripheral magnetic resonance imaging. Osteoarthritis Cartilage.

[13] Cicuttini F, Ding C, Wluka A, Davis S, Ebeling PR, Jones G (2005). Association of cartilage defects with loss of knee cartilage in healthy, middle-age adults: a prospective study. Arthritis Rheum.

[14] Swagerty DL, Jr. Jr., Hellinger D (2001). Radiographic assessment of osteoarthritis. Am Fam Physician.

[15] Cheng Y, Macera CA, Davis DR, Ainsworth BE, Troped PJ, Blair SN (2000). Physical activity and self-reported, physician-diagnosed osteoarthritis: is physical activity a risk factor?. J Clin Epidemiol.

[16] Alentorn-Geli E, Samuelsson K, Musahl V, Green CL, Bhandari M, Karlsson J (2017). The association of recreational and competitive running with hip and knee osteoarthritis: a systematic review and meta-analysis. J Orthop Sports Phys Ther.

[17] Sward L, Hellstrom M, Jacobsson B, Peterson L (1990). Back pain and radiologic changes in the thoraco-lumbar spine of athletes. Spine.

[18] Sward L, Hellstrom M, Jacobsson B, Nyman R, Peterson L (1991). Disc degeneration and associated abnormalities of the spine in elite gymnasts. A magnetic resonance imaging study. Spine (Phila Pa 1976.

[19] Dimitriadis AT, Papagelopoulos PJ, Smith FW (2011). Intervertebral disc changes after 1 h of running: a study on athletes. J Int Med Res.

[20] Reilly T, Chana D (1994). Spinal shrinkage in fast bowling. Ergonomics.

[21] Kingsley MI, D'Silva LA, Jennings C, Humphries B, Dalbo VJ, Scanlan AT (2012). Moderate-intensity running causes intervertebral disc compression in young adults. Med Sci Sports Exerc.

[22] Teraguchi M, Yoshimura N, Hashizume H (2014). Prevalence and distribution of intervertebral disc degeneration over the entire spine in a population-based cohort: the Wakayama Spine Study. Osteoarthritis Cartilage.

[23] Boden SD, McCowin PR, Davis DO, Dina TS, Mark AS, Wiesel S (1990). Abnormal magnetic-resonance scans of the cervical spine in asymptomatic subjects. A prospective investigation. J Bone Joint Surg Am.

